# Molecular Dynamics of Apolipoprotein Genotypes APOE4 and SNARE Family Proteins and Their Impact on Alzheimer’s Disease

**DOI:** 10.3390/life15020223

**Published:** 2025-02-02

**Authors:** Yuqing Wang, Xuefeng Liu, Pengtao Zheng, Qing Xie, Chenxiang Wang, Chaoyang Pang

**Affiliations:** 1 School of Physics, Chengdu University of Technology, Chengdu 610059, China; yuqingwang777@126.com (Y.W.);; 2 College of Computer Science, Sichuan Normal University, Chengdu 610101, China

**Keywords:** Alzheimer’s disease, synaptic dysfunction, apolipoprotein E, molecular dynamics simulations, SNARE complex, molecular mechanics Poisson–Boltzmann surface area

## Abstract

Alzheimer’s disease is a chronic neurodegenerative disorder characterized by progressive memory loss and a significant impact on quality of life. The APOE ε4 allele is a major genetic contributor to AD pathogenesis, with synaptic dysfunction being a central hallmark in its pathophysiology. While the role of APOE4 in reducing SNARE protein levels has been established, the underlying molecular mechanisms of this interaction remain obscure. Our research employs molecular dynamics simulations to analyze interactions between APOE4 and APOE3 isoforms and the synaptic proteins VAMP2, SNAP25, and SYNTAXIN1, which play crucial roles in the presynaptic membrane. Our findings reveal that APOE4 significantly destabilizes the SNARE complex, suppresses its structural dynamics, and reduces hydrogen bonding, consequently partially hindering neurotransmitter release—a very likely discovery for elucidating synaptic dysfunction in Alzheimer’s disease. We identified that APOE4 exhibits a diminished affinity for the SNARE complex in comparison to APOE3. This observation suggests that APOE4 may play a role in modulating the stability of the SNARE complex, potentially impacting the progression and occurrence of Alzheimer’s disease through free energy analysis. This work highlights the perturbations in synaptic function mediated by APOE4, which may offer novel insights into the molecular underpinnings of AD. By elucidating the molecular interplay between APOE4 and the SNARE complex, our study not only enhances our comprehension of AD’s synaptic pathology but also paves the way for devising innovative therapeutic interventions, such as targeting the APOE4–SNARE complex interaction or to restore neurotransmitter release.

## 1. Introduction

In China, approximately 16 million elderly individuals are affected by Alzheimer’s disease (AD) [1], a neurodegenerative disorder characterized by clinical dementia, significant memory decline, and distinct pathological features [2]. As one of the primary causes of cognitive decline in middle-aged and elderly populations, AD severely impairs patients’ social interactions and occupational abilities. The hallmark pathological features of Alzheimer’s disease include the formation of neurofibrillary tangles (NFTs), the intracellular and extracellular deposition of β-amyloid (Aβ), synaptic dysfunction, and neuronal death [3]. Notably, studies have identified significant synaptic structural and functional changes in the neocortex during the early stages of the disease, which persist throughout its progression until the patient’s death [4,5]. Clinical and pathological research further highlights a strong correlation between synaptic signal transmission deficits and the severity of memory impairment in AD patients [6,7]. These findings suggest that Alzheimer’s disease is fundamentally a synaptic dysfunction disorder, with its hallmark symptom—cognitive dysfunction—stemming from pathological damage to synaptic structure and function [8]. Given the limited efficacy of current treatment strategies, a deeper investigation into the specific pathophysiological mechanisms of Alzheimer’s disease—particularly those related to synaptic dysfunction and molecular interactions—remains essential.

**Figure 1:**
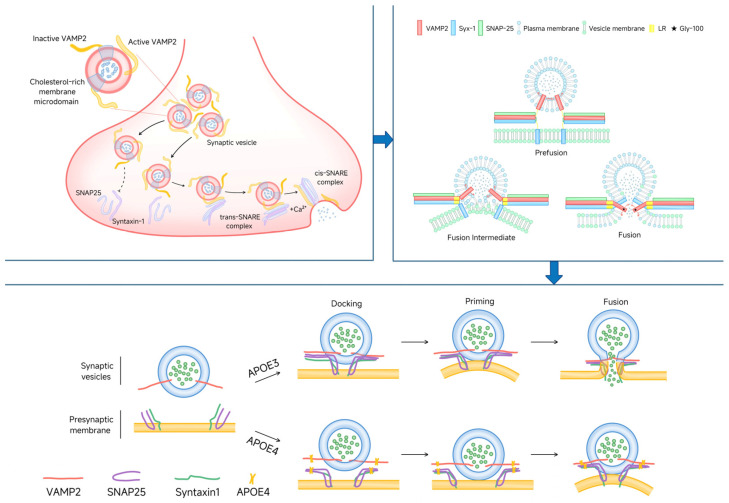
The upper section illustrates the role of the SNARE complex in the synaptic vesicle release process, while the diagram on the right provides a more detailed depiction of the three stages of vesicle fusion mediated by the SNARE complex. The lower section compares the effects of APOE3 and APOE4 on the function of the SNARE complex. APOE3 maintains SNARE complex stabilization and ensures vesicle docking and fusion to release neurotransmitters; in contrast, APOE4 disrupts the interaction of VAMP2 and SYNTAXIN1 with SNAP25 and impairs synaptic function.

The release of neurotransmitters is a critical step in neuronal communication, directly impacting cognitive function and the stability of neural networks within the brain [9,10]. As is cited in Figure 1, central to this process is the SNARE complex, composed of VAMP2, SNAP25, and SYNTAXIN1. VAMP2 resides on the vesicle membrane, while SNAP25 and SYNTAXIN1 are located on the presynaptic membrane [11,12]. Together, they cooperate to form a stable SNARE complex that facilitates the precise docking of vesicles with the presynaptic membrane and the efficient release of neurotransmitters. However, in the early stages of Alzheimer’s disease, this mechanism is often disrupted. The presence and accumulation of APOE4 can interfere with the normal assembly and function of the SNARE complex, resulting in structural damage and dysfunction [13]. This disruption impedes the release of neurotransmitters, such as acetylcholine, thereby impairing neural signaling and triggering memory loss, cognitive decline, and deficits in neuronal communication [14]. Consequently, SNARE complex dysfunction becomes a key hallmark of synaptic pathology in Alzheimer’s disease and a primary cause of impaired neurotransmitter release and the ensuing cognitive deficits.

Immunoprecipitation experiments have confirmed that APOE4 inhibits the assembly of SNARE complexes, with its presence resulting in a roughly 30% decrease in their numbers [15,16]. This reduction negatively impacts the release of synaptic vesicles, contributing to synaptic dysfunction and cognitive impairment. Subsequent research revealed that the synaptic-vesicle-membrane-associated protein VAMP2 can bind to both APOE3 and APOE4, but with a notably stronger affinity for APOE4. This binding disparity may significantly impair the efficiency of SNARE complex assembly, disrupt neurotransmitter release, and ultimately lead to compromised synaptic signaling [17]. Considering the pivotal role of APOE genotypes in the release of synaptic vesicles, a thorough examination into the molecular interactions and energetic shifts within the VAMP2/SNAP25/SYNTAXIN1 complex under APOE3 and APOE4 conditions is essential [18]. In this study, we employed dynamic simulation methods to systematically investigate the structural stability and functional attributes of the VAMP2/SNAP25/SYNTAXIN1 complex in the presence of (+)APOE4 and (+)APOE3. Our findings offer theoretical insights into the mechanisms by which APOE4 contributes to synaptic dysfunction in Alzheimer’s disease.

In our study, we employed kinetic simulation to uncover the intrinsic characteristics and interplay among five pivotal genes—VAMP2, SNAP25, SYNTAXIN1, and the APOE3/APOE4 isoforms. These kinetic analyses provided a comprehensive view of the coordination and interactions among these genes within the Alzheimer’s disease (AD) framework, aiding in the identification of significant alterations linked to disease progression. Our findings indicated that the correlation among VAMP2, SNAP25, SYNTAXIN1, and APOE genotypes underwent substantial changes with the advancement of AD, as demonstrated by heightened distributional disparities and notable oscillations in multiple eigenvalues. Utilizing molecular dynamics simulations, we conducted an in-depth analysis of the conformational feasibility, stability, and interaction profiles of the protein complex involving APOE3 and APOE4 genotypes in the brains of AD patients. The outcomes revealed that APOE4 significantly disrupts the stable assembly of VAMP2 with SNAP25 and SYNTAXIN1, thereby impeding, to a considerable extent, the neurotransmitter release process through the fusion of synaptic vesicles with the presynaptic membrane. The ε4 allele of the apolipoprotein E (APOE) gene is a predominant genetic risk factor for AD, with APOE4 being associated with detrimental effects on various neurodegenerative conditions linked to cognitive impairment, including AD.

Synthesizing a wealth of evidence, it has become evident that APOE4 may intensify disease progression via synaptic damage pathways. However, the precise molecular underpinnings have remained elusive. Our research contributes novel molecular evidence highlighting the critical role of APOE4 in synaptic dysfunction and offers valuable insights for the development of targeted therapeutic strategies for Alzheimer’s disease.

## 2. Materials and Methods

### 2.1. Processing the Crystal Structures of Selected Proteins

In this study, we obtained the three-dimensional structure of APOE3 from the RCSB Protein Data Bank (PDB ID: 2L7B). The PDB ID for the VAMP2/SYNTAXIN1/SNAP25 (SNARE) complex is 1N7S [19]. APOE3 is a homologous allele of APOE4, with a difference in the residue at position 112. Specifically, APOE3 contains a cysteine (Cys) residue at this position, which plays a key role in the protein’s conformation and function [20,21]. Previous studies have shown that mutating Cys112 to arginine (Arg) introduces a positive charge, potentially altering the local hydrogen bonding network and the electrostatic surface properties. This structural change may affect the overall ligand-binding ability of the protein [22,23]. In order to obtain an accurate structure of APOE4 through homology modeling, SWISS-MODEL Workspace and PyMOL2.5—two software packages characterized respectively by rapid generation ability of high-quality models and powerful capabilities in structural analysis and evaluation—were employed to generate and analyze the structure. After comparison, the model with the least impedance and best structural quality was selected and exported as APOE4. A molecular docking analysis was subsequently performed [24]. Before performing molecular docking, the target protein was prepared by eliminating alternative conformations, adjusting terminal residues, and correcting bond orders to ensure its structural integrity [25,26]. All steps were carried out using the “Build Side Chain” module of SPDBV_4.1 software [27]. For a detailed description of the procedure, please refer to the Supplementary Materials.

### 2.2. Molecular Docking

We optimized the energies of the target proteins and ligands using a CHARMM-based smart minimization method to ensure the structures reached their lowest energy states before the molecular docking stage, providing a solid foundation for subsequent analyses. Rigid docking studies were then conducted using PyDOCK3.0 [28]. During the docking process, we employed PyDOCK’s default parameter set, which includes potential energy, desolvation energy, and van der Waals energy as the scoring criteria. To enhance the reliability and reproducibility of the results, we also consulted parameter optimization strategies from similar studies in the literature. The best docking results were determined based on the highest overall score, taking into account the involvement of key amino acids and the functional relevance of the binding site. After docking, we visualized the results using AutoDock4.2.6 to analyze the ligand-binding site and conformation within the protein [29]. Docked conformations were scored, and those with higher scores underwent a clustering analysis to identify those with the lowest binding energy. A lower binding energy indicates a stronger interaction between the ligand and receptor protein. Finally, we performed molecular dynamics (MD) simulations on the selected best-binding complexes to assess the stability of the protein-ligand interactions under simulated in vitro and in vivo conditions [30].

### 2.3. Molecular Dynamics (MD) Simulation

In this study, molecular dynamics (MD) simulations were performed using the GROMACS 2023.5 software package at 310 K (37 °C) with a GROMOS96 54A7 force field. The 54A7 force field optimizes torsional angles, stabilizes α-helices, and adjusts non-bonded interactions to enhance simulation accuracy, and it is well-validated in similar systems, making it ideal for short-time protein dynamics studies [31]. Prior to the simulations, the complex structures were processed using PyMOL2.5 to remove initial solvents and ions. Simulations were performed over a timescale of 100 ns for each system. All systems were solvated using the Simple Point Charge (SPC) water model. A cubic water box extending 5 Å from the protein surface was created, and five ${Na}^{+}$ and five ${Cl}^{+}$ ions were added to neutralize the system. Long-range electrostatic interactions were computed using the Particle Mesh Ewald (PME) method, and energy minimization was carried out using the steepest descent method with 50,000 steps [32]. All simulations were conducted under periodic boundary conditions using the NVT and NPT ensembles. During position-restrained MD simulations, the temperature (310 K) and pressure (1 bar) were maintained using a V-rescale thermosta for temperature control and a Berendsen barostat for pressure control [33,34]. Subsequently, four 100 ns MD simulations were performed for each complex, and the trajectories were averaged for analysis. Electrostatic interactions were calculated using the PME algorithm with a 2 fs time step [35], and all bond lengths were constrained using the LINCS algorithm. The final trajectory data were visualized using VMD [36].

### 2.4. Dynamical Motion Analysis

We used a rough network-based model to analyze the association dynamics between different residues. First, we defined the nodes. In this network, each amino acid is considered as a node and these nodes are located on the Cα atoms of different amino acids [37]. The next step was to define the edges between the coarse-grained nodes. Based on the interactions between the residues of the protein complex, the complex folds into a specific functional structure [38].

Data on the movement of the proteins were obtained by molecular dynamics simulations [39]. These data typically include the position of each atom over time during the simulation, and the motion of the system is determined by the Bio3d plug-in for the R language package [40,41], which calculates dynamic network correlations between individual residues. Protein motion data were obtained by reading molecular dynamics simulation trajectory files (e.g., PDB and DCD files). These trajectories were then aligned to eliminate overall translational and rotational effects to ensure accuracy for subsequent analysis. Next, the fluctuations of each atom were calculated, specifically, the deviation of each atom relative to its average position [42]. Using this fluctuation data, the dynamic cross-correlation between each pair of atoms was calculated to generate a DCCM matrix. The elements of this matrix represent the kinematic correlations between each pair of atoms. Finally, the DCCM matrix was visualized by a heatmap or color map to visualize the dynamic correlations within the protein [43].

In the analysis process, the degree of correlation motion between two residues (or two atoms) can be expressed by the cross-correlation coefficient $C_{i,j}$, which is defined as follows,

(1) $$C_{i,j}=\frac{\langle \delta {\vec{r}}_{i}\left(t\right)\cdot \delta {\vec{r}}_{j}\left(t\right)\rangle }{\sqrt{\langle (\delta {\vec{r}}_{i}(t){)}^{2}\rangle \langle (\delta {\vec{r}}_{j}(t){)}^{2}\rangle }}$$

where $C_{i,j}$ represents the kinematic correlation between atoms i and j, and $\langle \delta {\vec{r}}_{i}(t)\cdot \delta {\vec{r}}_{j}(t)\rangle$ denotes the dot-product time averaging between atoms i and j, and the value of $C_{i,j}$ ranges from −1 to 1. If the closest distance is less than 4.5 Å, then the contact diagram is expressed as two residues in contact with each other.

### 2.5. MM-PBSA Calculations

Molecular Mechanics Poisson–Boltzmann Surface Area (MM-PBSA), which is assessed by the AMBER toolbox and the MMPBSA.py script, was used to compute the binding free energy of protein–protein complexes ($∆G_{bind}$) [44]. One of the most popular techniques for determining the interaction energies of biomolecular complexes is the MM-PBSA approach. Important conformational changes and entropic contributions to binding energies can be decoded using MM-PBSA in conjunction with MD simulations [45]. In this work, MM-PBSA with energy splitting was used to calculate the binding energies between SNARE complexes in three systems. Generally speaking, the free energy of protein and solvent binding ($∆G_{bind}$) can be written as follows:

(2) $$∆G_{bind}={∆E}_{MM}+∆G_{solv}-T∆S$$

and

(3) $$∆E_{MM}={∆E}_{bonded}+{∆E}_{vdw}+{∆E}_{ele}$$

(4) $$∆G_{solv}={∆G}_{polar}+{∆G}_{non-polar}$$

where ${∆G}_{bind}$ is the total free energy of the protein–protein complex, $∆E_{MM}$ denotes the molecular mechanics energy, which contains the energies of bonds, angles, dihedral angles, van der Waals energy, and electrostatic energy, $∆G_{solv}$ consists of the free energy of polar solvation and the free energy of non-polar solvation, and T∆S is the change in the entropy of the degrees of freedom before and after the molecule conformation.

## 3. Results

### 3.1. Rationalization of Protein Structure Selection and Docking Model Conformation

**Figure 2:**
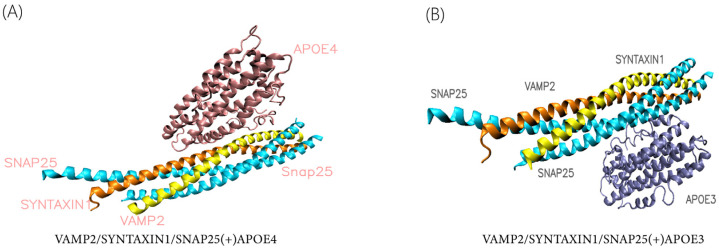
Docking protein complex (**A**) VAMP2/SYNTAXIN1/SNAP25(+)APOE4, (**B**) VAMP2/SYNTAXIN1/SNAP25(+)APOE3. Among them, the pink one is APOE4, the orange one is SYNTAXIN1, the yellow one is VAMP2, the blue one is SNAP25, and the dark blue one is APOE3.

We screened APOE3 (PDB ID:2L7B) and VAMP2/SNAP25/SYNTAXIN1 (SNARE PDB ID:1N7S) from the RSCD PDB Protein Data Base by examining the resolution, R-value and R-free value, B-factor, and integrity of various structures of proteins of the same sequence. After replacing APOE3′s residue 112 with PyMOL2.5, the homologous protein APOE4 was produced. PyDOCK3.0 was then used to rigidly dock APOE3 and APOE4 with the VAMP2–SNAP25–SYNTAXIN1 complex, respectively. We confirmed that the protein complexes produced by docking were conformationally sound. Two helpful metrics for comprehending the structural characteristics of proteins are phi and psi. By examining the two rotational angles of the α-carbon of the phi (C-N bond) and psi (C-C bond) in the protein complexes, the quality of the model of the docking results as well as the structural soundness were assessed. The Ramachandran Plot is the name given to the plots of phi versus psi. The characteristics of amino acids or secondary structural elements of proteins are reflected in certain sections of Figure S1, whereas other regions are regarded as off-limits. Structural transitions may be shown by variations in phi/psi over time. We evaluated the quality of the complicated protein model in order to confirm the validity of the protein structure during docking. In our protein model, amino acid residues from the permissive and maximal permissive areas made up almost 97% of the entire protein. The final result is shown in Figure 2. This indicates that the docking model we produced can be applied to further molecular dynamics simulations and studies.

### 3.2. Stability of VAMP2/SYNTAXIN1/SNAP25(+)APOE4/(+)APOE3

**Figure 3:**
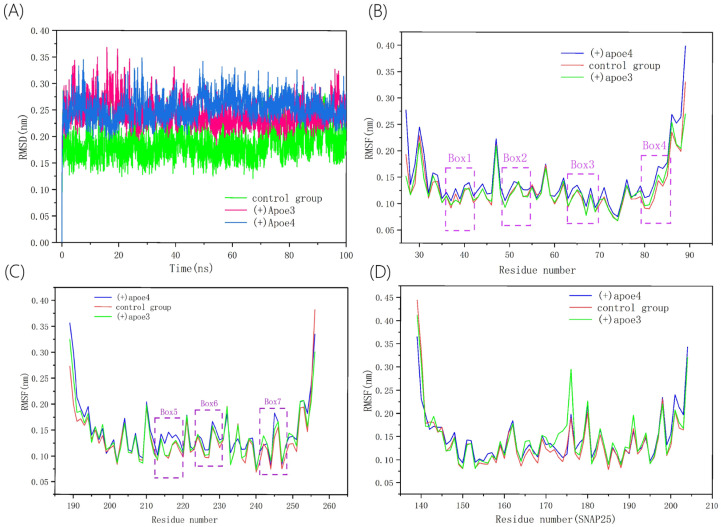
(**A**) Root-mean-square deviation (RMSD) of the Cα atoms of SNARE in the control and the two protein complexes. (**B**–**D**) Root-mean-square fluctuation (RMSF) corresponding to VAMP2, SYNTAXIN1, and SNAP25, respectively, where Box 1 to Box 7 denote regions with significant RMSF fluctuations under (+)APOE4 conditions, which correspond to the approximate residue ranges of 33–43, 47–54, 61–69, and 79–85, respectively, and 210–220, 225–230, and 240–248, respectively.

In our study, we performed an in-depth analysis of the molecular dynamics (MD) simulation data for the VAMP2/SYNTAXIN1/SNAP25 (SNARE) complex to evaluate its stability during engagements with VAMP2/SYNTAXIN1/SNAP25 complexes associated with either APOE4 or APOE3 isoforms [46]. The stability was assessed by monitoring the root-mean-square deviation (RMSD) values of the individual atoms within the complex, which were determined by the average distance between the overlapping protein backbone atoms. In the initial simulation phase (0–40 ns), the RMSD values for the VAMP2/SYNTAXIN1/SNAP25(+)APOE4 and VAMP2/SYNTAXIN1/SNAP25(+)APOE3 complexes were quite similar, potentially due to significant structural changes resulting from interactions with the surrounding solvent. RMSD values for all three protein complexes reached a plateau and showed minimal fluctuations in the last 50 ns of the simulation, indicating that the system had reached an equilibrium and the simulation had converged. The VAMP2/SYNTAXIN1/SNAP25(+)APOE4 complex had an average RMSD value of 0.253 nm, whereas the control group and the VAMP2/SYNTAXIN1/SNAP25 (+)APOE3 complex had values of 0.184 nm and 0.243 nm, respectively. Throughout the latter half of the simulation, the RMSD values for the VAMP2/SYNTAXIN1/SNAP25 (+)APOE3 complex and the control group were consistently lower than those for the VAMP2/SYNTAXIN1/SNAP25(+)APOE4 complex. The RMSD values indicated that APOE4 binding leads to increased fluctuations within the SNARE complex. To further analyze the atomic variations within the protein relative to their average positions, we calculated the root-mean-square fluctuation (RMSF) of the SNARE complex across the three simulations (Figure 3B). The RMSF of the SNARE complex containing APOE4 was distinct from that of the APOE4-free SNARE complex, with the VAMP2/SYNTAXIN1/SNAP25(+)APOE4 complex showing a notably higher RMSF. The residues indicated in Boxes 1–4 of Figure 3B and Boxes 5–7 of Figure 3C demonstrated heightened instability in the presence of (+)APOE4.

### 3.3. Hbond Number and Radius of Gyration in VAMP2/SYNTAXIN1/SNAP25

**Figure 4:**
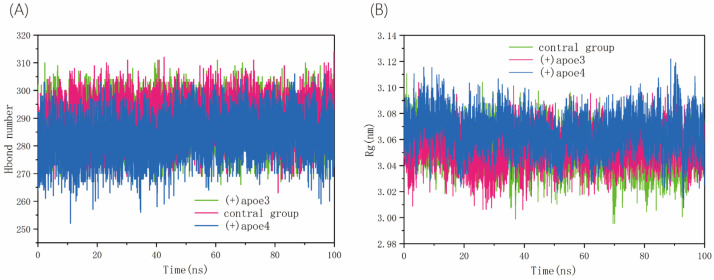
(**A**) Number of Hbonds of SNARE in protein complexes in control and both other groups; (**B**) radius of gyration.

While the number of hydrogen bonds can somewhat reflect the structural stability, folding state, and functional activity of proteins, hydrogen bonds are crucial for preserving protein stability and achieving their functions. They are also a key mechanism for indicating the strength of protein interactions [47]. One crucial metric for characterizing a protein molecule’s compactness or folding state is its radius of gyration. Thus, as illustrated in Figure 4, we examined the radius of gyration and the number of hydrogen bonds between VAMP2/SYNTAXIN1/SNAP25 in order to determine whether the addition of APOE4 influences their binding. While (+)APOE4′s radius of gyration greatly expanded during the simulations, the number of SNARE protein hydrogen bonds significantly decreased (Figure 4A). We also studied the distance and angular distribution of hydrogen bonds and the average period of existence to show the reality of the hydrogen bonds found during the simulations. The results showed that the hydrogen bonds formed were stable and effective because the distributions were narrower, the average angular distributions were smaller, and the overall distances of the hydrogen bonds were shorter. The hydrogen bond distances and angular distributions showed that the hydrogen bonds were comparatively stable throughout the three simulated systems. In addition, as shown in Figure 4B, the radius of gyration (Rg) was calculated to assess the protein’s overall compactness. A larger Rg, associated with fewer hydrogen bonds, suggests a looser and less stable structure. For the SNARE complex, an increased Rg implies greater flexibility and potentially compromised structural integrity. This flexibility may directly impact synaptic function, contributing to dysfunction by disrupting the precise interactions required for efficient neurotransmitter release.

### 3.4. Dynamic Cross-Correlation Matrix (DCCM) of VAMP2/SYNTAXIN1/SNAP25

**Figure 5:**
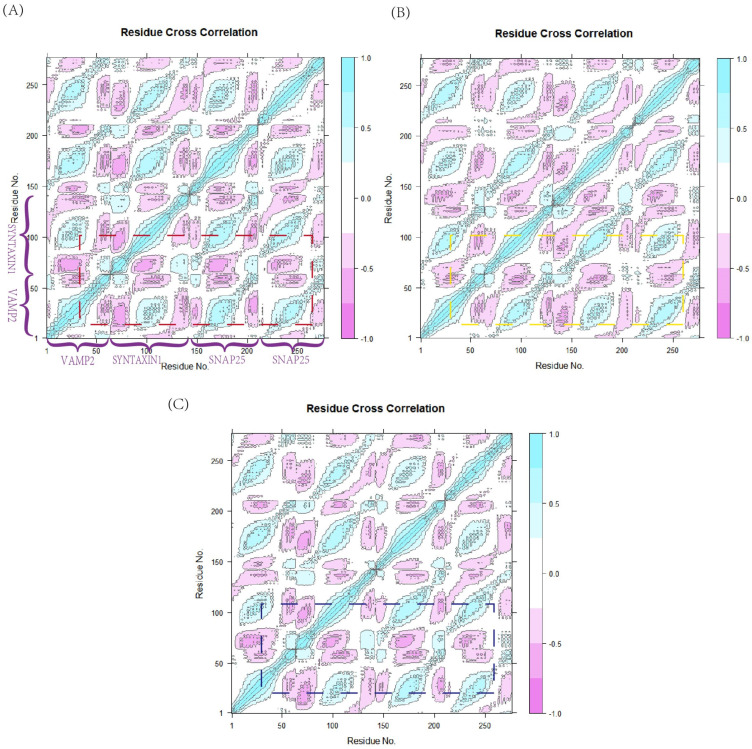
(**A**–**C**) Dynamic Cross−Correlation Matrix of the SNARE complex system representing (+)APOE4, the control group, and (+)APOE3, respectively.

We employed a dynamic correlation matrix analysis to investigate the functional displacements of SNARE complexes over time, aiming to gain deeper insights into how APOE4 decreases SNARE stability and to identify potential functional sites or critical structural domains [48]. For the correlation analysis, stable MD simulations with durations ranging from 50 to 70 ns were used. The correlation values were calculated by measuring the distance between any two residues in a 20 ns trajectory. If the distance was less than 4.5 Å in 75% of the frames, the correlation value was considered constant; otherwise, it was assumed to be zero. A positive correlation was defined when two residues moved together in the majority of frames, resulting in a positive correlation value, while a negative correlation was observed when residues moved in opposite directions in most frames. If the correlation coefficient between two residues was close to zero, their motions were considered independent. The results of the dynamic correlation analysis of the SNARE interaction interface are shown in the figure. The magnification and deepening of the red areas in Figure 5A,B indicate that the correlation between VAMP2 and SYNTAXIN1 and SNAP25 residues in the (+)APOE4 or (+)APOE3 complexes was lower compared to those in the control group in Figure 5C. By combining these results with the RMSF fluctuations of the residues and using PyMOL2.5 visualization software, we identified the following residues interacting with the SNARE protein complex: residues 50-60 of VAMP2, 210-235 of SYNTAXIN1, and 145-155 of the SNAP25/2 chain. In the (+)APOE4 complex, residues in VAMP2 exhibited low dynamic correlation with residues in SYNTAXIN1 and SNAP25. Interestingly, when comparing Figure 5A,B, the dynamic correlation of the (+)APOE4 protein complex was found to be significantly lower than that of the (+)APOE3 complex.

### 3.5. The Binding Free Energy of SNARE

**Figure 6:**
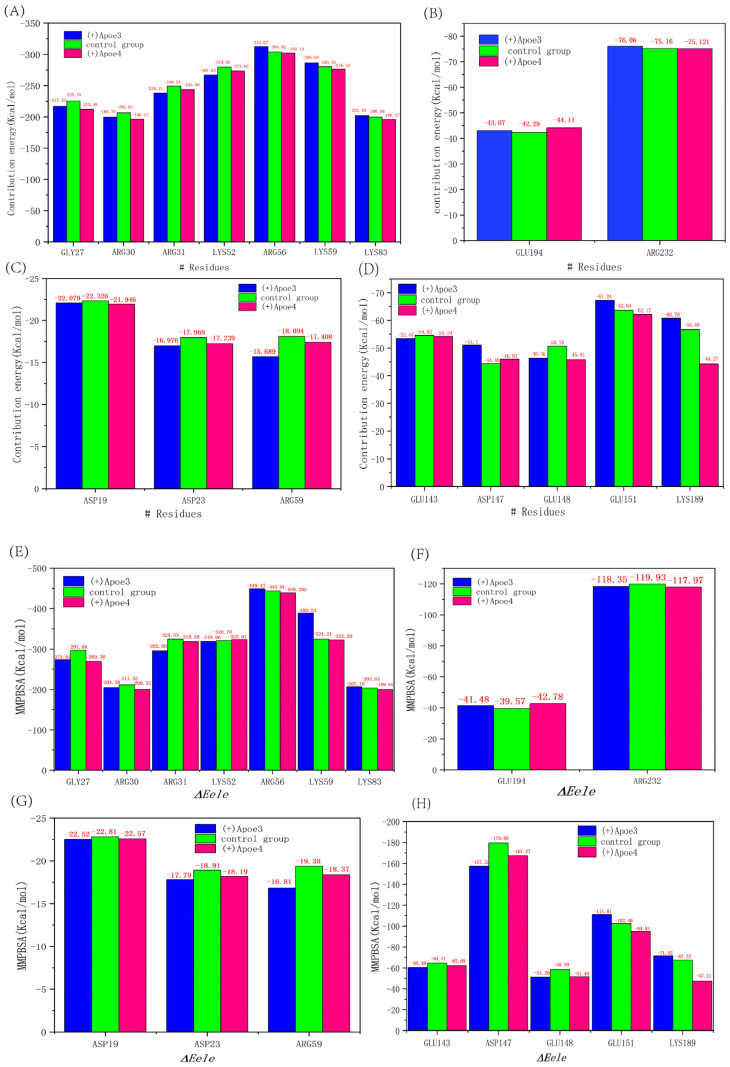
(**A**,**B**) shows the residue energy contributions of VAMP2 and SYNTAXIN1, respectively; (**C**,**D**) shows the residue energy contributions of the first and second strands of SNAP25 ibid; and (**E**–**H**) shows the electrostatic interaction analyses of the corresponding proteins. The energy decomposition of each residue was calculated based on the 50–70 ns MD trajectory.

To elucidate the binding process for each amino acid, we utilized gmx_MMPBSA to compute the binding free energies (BFEs) between 50 ns and 70 ns during the molecular dynamics (MD) simulations. Subsequently, we conducted energy decomposition analyses for each amino acid at the SNARE interaction sites [49,50]. We determined the total binding free energy (BFE) for the simulated complexes over the observation period and found that the control group exhibited a BFE of −503.81 kJ/mol. In contrast, the VAMP2/SYNTAXIN1/SNAP25(+)APOE4 complex had the lowest BFE at −372.112 kJ/mol, with the VAMP2/SYNTAXIN1/SNAP25(+)APOE3 complex trailing closely at −408.935 kJ/mol (Table 1). These results indicated that the VAMP2/SYNTAXIN1/SNAP25(+)APOE4 complex possesses the weakest binding affinity among the SNARE complexes, culminating in the formation of an unstable complex. The trio of simulations revealed that the van der Waals interactions, electrostatic forces, and solvation free energy constitute the principal determinants of the binding energy within the SNARE complex. A thorough analysis of the energetics pinpointed the crucial amino acids involved in the binding process. We employed an energy partitioning methodology to quantify the energetic contribution of each amino acid. Sequentially selecting the key-acting residues from each of the four chains is theoretically sound, as the residues with significant contributions are expected to exhibit high absolute values of $∆E_{total}$. For VAMP2, the pivotal residues are Gly27, Arg30, Arg31, Lys52, Arg56, Lys59, and Lys83; for SYNTAXIN1, they are Glu194 and Arg232; and for SNAP25, they are Glu143, Asp147, Glu148, Glu151, and Lys189 (Figure 6A–D). The differences in binding free energy were more pronounced for the (+)APOE4 complex in SNAP25 residues compared to the (+)APOE3 complex, albeit with a slightly smaller absolute value. To delve deeper into the fluctuations of binding free energy, an examination of the data across the three systems revealed that the $∆E_{ele}$ of the pivotal residues within the SNARE complex’s tetrachain was marginally elevated in the control group when compared to the other groups. Within the (+)APOE4 SNARE complex system, the absolute value of $∆E_{ele}$ was found to be slightly diminished relative to the other groups, suggesting that these key residues exert a weaker interactive force. Electrostatic interactions, stemming from the mutual attraction or repulsion among charged residues, significantly influence the thermodynamic aspects of protein folding and ligand binding. Through a more granular analysis of the electrostatic components, we were able to pinpoint and quantify the extent to which charge distribution contributes to the binding free energy.

## 4. Discussion

The APOE genotype, a significant genetic risk factor of Alzheimer’s disease, has been shown to interact with VAMP2. However, the precise impact of APOE4 on the molecular dynamics and conformational stability of the SNARE complex, particularly in the early stages of AD, remains unclear [51]. To address this, we conducted molecular dynamics simulations on SNAP25, VAMP2, and SYNTAXIN1, successfully identifying the target proteins with high precision, resulting in robust and reliable simulation outcomes. We established experimental groups, labeled as VAMP2/SYNTAXIN1/SNAP25(+)APOE4, VAMP2/SYNTAXIN1/SNAP25(+)APOE3, and a control SNARE group, for further molecular dynamics simulations. This selection was made to scrutinize the influence of the APOE genotype on the modulation of the VAMP2/SYNTAXIN1/SNAP25 protein complex system within the brains of individuals with Alzheimer’s disease. The protein regulatory system formed by VAMP2/SYNTAXIN1/SNAP25(+)APOE4 may indirectly contribute to the development of Alzheimer’s disease through the in vivo regulation of Alzheimer’s disease patients’ progression, according to a comparison of the simulation results of VAMP2/SYNTAXIN1/SNAP25(+)APOE4 and VAMP2/SYNTAXIN1/SNAP25(+)APOE3. APOE4 is believed to be a key protein in this system.

According to the estimated RMSD values, the control SNARE and protein complex system (+)APOE3 displayed a more stable condition, while (+)APOE4 markedly enhanced the fluctuation of SNARE. This implies that the structural alterations and affinity of the VAMP2/SYNTAXIN1/SNAP25 complex may be diminished by the interaction of APOE4 with VAMP2. The Box 1–Box 7 region delineated in Figure 4B precisely captures the substantial reduction in SNARE complex stability exerted by the (+)APOE4 system. The residue diversity illustrated within the boxes indicates that both APOE3 and APOE4 increase the variability of key residues, thereby weakening intra-SNARE protein interactions. However, APOE4 exerts a more pronounced impact on the SNARE complex compared to APOE3. Both APOE isoforms reduce the number of hydrogen bonds between VAMP2 and SYNTAXIN1 as well as SNAP25, but the reduction caused by APOE4 is significantly more substantial. A dynamic cross-correlation analysis further revealed that the (+)APOE4 system disrupts interfacial stability among SNARE proteins to a greater extent than the APOE3 system. APOE4 exhibits more fluctuations, likely due to its structural instability and increased flexibility, which further exacerbates the uncertainty of SNARE protein interactions. In contrast, while APOE3 also induces some degree of fluctuation, it maintains noticeably greater stability compared to APOE4. These findings highlight the potentially more detrimental role of APOE4 in the pathogenesis of Alzheimer’s disease.

We subsequently analyzed the data presented in Table 1 to gain a deeper comprehension of the mechanisms by which the (+)APOE4 protein complex regulates the VAMP2/SYNTAXIN1/SNAP25 interaction. The computed binding free energy (BFE) of the protein complex encompasses various components, including van der Waals energy, electrostatic energy, and polar solvation energy. Negative values in the table signify favorable interactions, while positive values denote unfavorable ones. Employing the MMPBSA methodology, we calculated the binding free energy (BFE) between VAMP2/SYNTAXIN1/SNAP25, focusing on the interaction residues within the SNARE complex. The group with (+)APOE4 exhibited a notably enhanced fluctuation compared to the other two groups, indicating a significant impact on the stability of the SNARE complex, particularly in terms of hydrogen bonding and conformational changes. It seems that the VAMP2/SYNTAXIN1/SNAP25 complex in association with (+)APOE4 undermined its own structural integrity by diminishing the binding affinity between the SNARE components. This observation is consistent with our previous findings that the presence of (+)APOE4 can adversely affect the stability of the SNARE molecular structure. A further energy decomposition analysis elucidated the energetic contributions of each amino acid. This helped to reveal which specific amino acids play important roles in the stability, binding affinity, and functionality of the protein complex, and the absolute value of $∆E_{total}$ was employed to identify critical residues that exert a significant influence on the binding process. Our research identified critical residues at the binding interface, including the amino acid residues Glu194 and Arg232 on SYNTAXIN1, Gly27, Arg30, Arg31, Lys52, Arg56, Lys59, and Lys83 on VAMP2, and Glu143, Asp147, Glu148, Glu151, and Lys189 on SNAP25, which play pivotal roles in the binding process. Most of these critical residues are positively charged, exhibiting strong electrostatic interactions. The critical residues of VAMP2 exhibit an average $∆E_{total}$ exceeding 200 kcal/mol, in contrast to the 50–60 kcal/mol observed for those of SYNTAXIN1 and SNAP25, which are considerably lower. This disparity suggests that the essential residues of VAMP2 fulfill a more substantial role within the SNARE complex system. The stability of the SNARE complex system is significantly impacted by these essential residues, particularly those at positions 52, 56, and 59, which may even be crucial for its separation. Biomolecules are typically charged, and the attraction or repulsion between charges directly affects the binding affinity. Analyzing the electrostatic energy contributions of residues helps to further identify key amino acids. With $∆E_{ele}$ values as high as −300 kcal/mol or higher, the three SNARE systems exhibit strong electrostatic contacts at the important residues. More intriguingly, the (+)APOE4 group exhibited a significantly larger differential in $∆E_{ele}$ across the residues of the SNARE protein complex compared to both (+)APOE3 and the control group (Figure 6B). Interestingly, the (+)APOE4 group also demonstrated lower ∆Eelec values for residues on VAMP2 and the associated protein complexes, distinguishing it from the other two groups. These findings also reveal that APOE4 serves as a significant inhibitor of protein complex systems, particularly in the context of modulating electrostatic interactions. Given that electrostatic energy is the predominant force governing the interaction between SNARE residues, it plays a crucial role in the binding process. Nevertheless, the presence of APOE4 adversely affects the binding capacity, as evidenced by a substantial reduction in the $∆E_{ele}$ of critical residues within the VAMP2/SYNTAXIN1/SNAP25 assembly of the SNARE complex. This interaction has profound effects. By diminishing the BFE, APOE4 effectively inhibits the convergence of the three SNARE protein strands, as inferred from a comparative analysis of APOE genotypes. In addition to directly affecting SNARE complex stability, APOE4 may accelerate the progression of Alzheimer’s disease through the activation of neuroinflammatory responses. APOE4 leads to microglial hyperactivation and promotes the accumulation of β-amyloid, which in turn impairs synaptic function and exacerbates cognitive decline. In addition, APOE4 gene load has a significant effect on synaptic function, and in particular, APOE4 heterozygotes (E4/E4) exhibited more significant synaptic damage and cognitive decline compared to APOE4 heterozygotes (E3/E4) or APOE3 heterozygotes (E3/E3). These differences may be related to the existence of different mechanisms in protein expression and metabolism in APOE4 purists and heterozygotes. For example, APOE4 pure heterozygotes are usually accompanied by stronger β-amyloid accumulation and neuroinflammatory responses, whereas APOE4 heterozygotes still maintain higher synaptic function in some cases.

## 5. Conclusions

In conclusion, the present study delved into the effects of APOE4 on the VAMP2/SYNTAXIN1/SNAP25 complex through molecular dynamics simulations, revealing the mechanism by which it may contribute to early synaptic dysfunction in Alzheimer’s disease (AD). The results showed that (+)APOE4 significantly weakened the stability and enhanced the dynamic fluctuation of the SNARE complex compared to APOE3, which was mainly due to the reduction in the number of hydrogen bonds and the disruption of SNARE protein-binding interface interactions. Energetic decomposition analyses revealed that key residues in VAMP2 (e.g., Arg56 and Lys59), which play a crucial role in the complex binding process, are more susceptible to disruption in the presence of APOE4. In addition, a dynamic cross-correlation analysis further revealed that APOE4 significantly reduces the stability of the SNARE complex interface by increasing structural flexibility and residue variability, thereby exacerbating the uncertainty of protein–protein interactions. These simulation data further validate the findings from immunoprecipitation experiments that the expression and assembly of SNARE proteins are reduced by approximately 30% in the AD mouse model expressing APOE4. The present study systematically reveals the mechanism by which APOE4 accelerates the pathological process of AD by modulating the molecular dynamics and interaction network of SNARE proteins. Finally, in the future, by screening small molecule compounds or peptide inhibitors targeting key residues of VAMP2 (e.g., Arg56 and Lys59) or stabilizing the SNARE complex, we may evaluate their ameliorative effects on synaptic functions and AD pathological processes in cellular and animal models. It may provide new therapeutic strategies for early intervention in AD.

## Figures and Tables

**Table 1:** The binding free energy was decomposed to a van der Waals interaction (${∆E}_{vdw}$), electrostatic interaction ($∆E_{ele}$), and polar solvation energy (${∆G}_{solv,PB}$). Energies are in kcal mol^−1^.

kcal mol^−1^	${∆G}_{total}$	${∆E}_{vdW}$	${∆E}_{ele}$	${∆G}_{solv}$
Control group	−503.810	−1062.276	−1256.585	1815.051
(+)APOE3	−408.935	−1084.457	−1225.323	1900.845
(+)APOE4	−372.112	−1074.133	−1190.196	1892.216

## Data Availability

Data are available from the corresponding author upon reasonable request.

## Supplementary Materials

The following supporting information can be downloaded at: https://www.mdpi.com/article/10.3390/life15020223/s1, Figures S1 and S2: Ramachandran plots; Figures S3–S5: the binding free energy values of key residues; Figure S6: the specific locations where key residues exert their effects; Figure S7: The predicted structure diagram of APOE4; Figure S8: The specific values of RMSF in each box from Box 1 to Box 7.
