# Supplementary material for: Molecular Dynamics of Apolipoprotein Genotypes APOE4 and SNARE Family Proteins and Their Impact on Alzheimer’s Disease
Source: Life (Basel). 2025 Feb 2;15(2):223. doi: 10.3390/life15020223 (PMC11855958; doi:10.3390/life15020223)
Supplement: Supplementary file 1 [file life-15-00223-s001.zip › life-3424914-supplementary.pdf]

supplementary material

(S1) (S2)

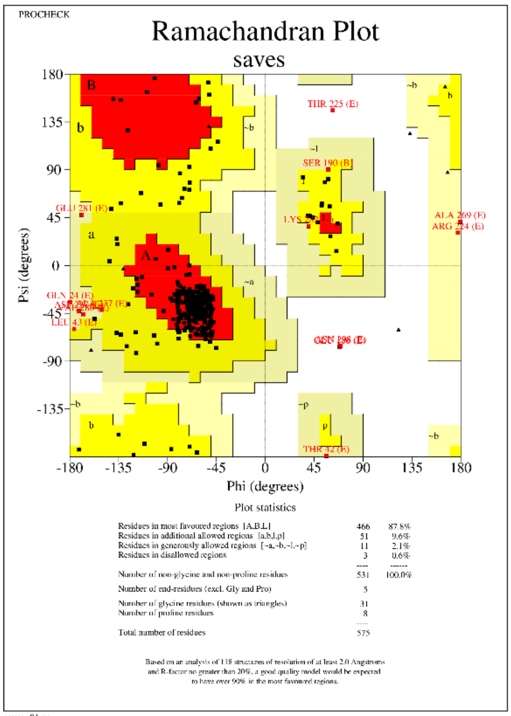

saves_01.ps

(+)APOE4

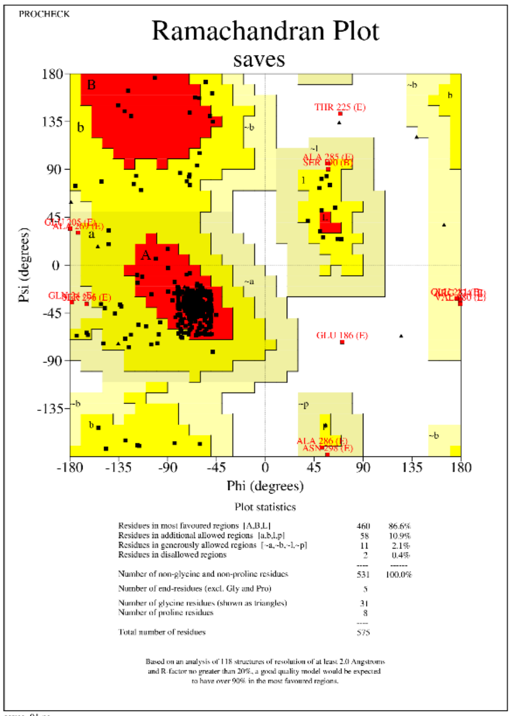

saves_01.ps

(+)APOE3

Fig.**S1** and **S2** represent the Ramachandran plots for the (+)APOE4 and (+)APOE3 complexes, respectively. In these plots, the red and yellow regions denote areas that must be entered for the proper functioning of the complexes, while all other areas are considered inaccessible or non-enterable regions. These designated areas are crucial for understanding the dynamic behavior and stability of the APOE isoforms within the context of their biological interactions and potential therapeutic implications.

(S3)

(VAMP2) ΔG_{total}	GLY27	ARG30	ARG31	LYS52	ARG56	LYS59	LYS83
APOE4	-212.38	-196.37	-243.8	-273.66	-302.13	-276.52	-196.17
control group	-225.7	-206.82	-249.54	-279.85	-304.02	-280.33	-202.49
APOE3	-217.32	-199.7	-238.21	-267.05	-312.67	-286.64	-200.08

(S4)

(SNAP25) ΔG_{total}	ASP19	ASP23	ARG59	GLU143	ASP147	GLU148	GLU151	LYS189
APOE4	-21.946	-17.239	-17.408	-54.19	-46.03	-45.81	-62.17	-44.27
control group	-22.326	-17.969	-18.094	-54.62	-44.4	-50.76	-63.64	-56.68
APOE3	-22.079	-16.976	-15.689	-53.44	-51.1	-46.36	-67.24	-60.78

(S5)

(SYNTAXIN1) ΔG_{total}	GLU194	ARG232
APOE4	-44.11	-75.121
control group	-42.29	-75.16
APOE3	-43.07	-76.06

Fig.**S3, S4**, and **S5** respectively illustrate the binding free energy values of key residues for VAMP2, SNAP25, and SYNTAXIN1. These data provide insightful views into the interaction energies of these key residues within the proteins, thereby aiding in the understanding of their functions and stabilities within the complex.

S6

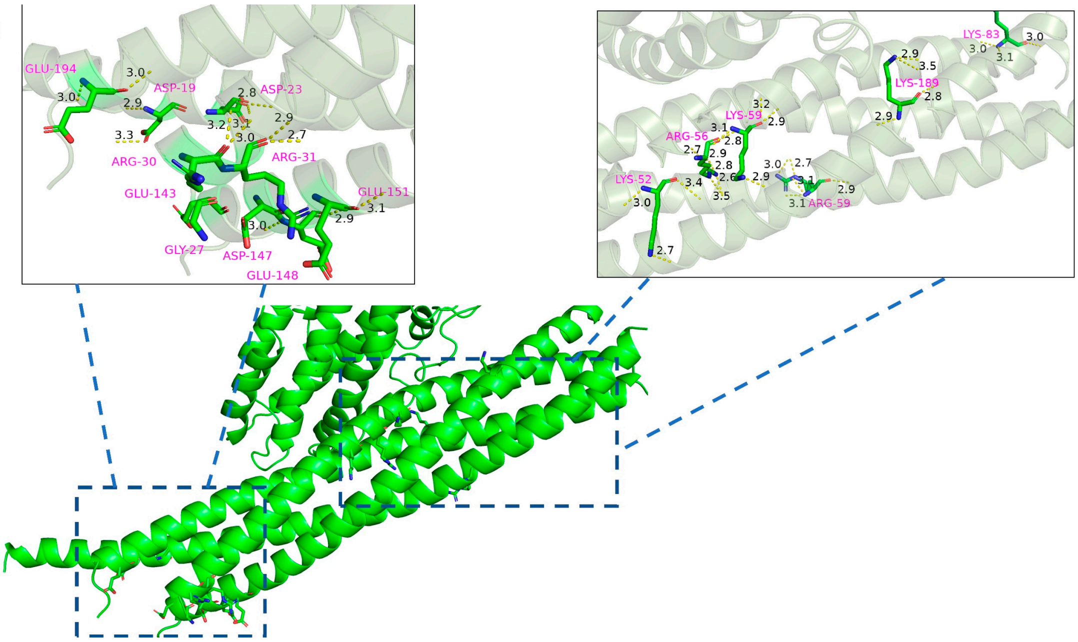

Fig.S6 The revised figure presents an analysis of residue interactions, pinpointing the specific locations where key residues exert their effects.

APOE3

KVEQAVETEPEPELRQQTEWQSGQRWELALGRFWDYLRWVQTLSEQVQEELLSSQVTQELRALMDETMKELKAYKSELEE

ARLSKELQAAQARLGADMEDVCGRLVQYRGEVQAMLGQSTEELRVRLASHLRKLRKRLLRDADDLQKRLAVYQAGAREGA

112

ERGLSAIRERLGPLVEQGRVRAATVGSLAGQPLQERAQAWGERLRARMEEMGSRTRDRLDEVKEQVAEVRAKLEEQAQQI

RLQAEAAQARLKSRFEPLAEDMQRQWAGQVEKVQAAEGTSAAPVPSDNH

APOE4

KVEQAVETEPEPELRQQTEWQSGQRWELALGRFWDYLRWVQTLSEQVQEELLSSQVTQELRALMDETMKELKAYKSELEE

ARLSKELQAAQARLGADMEDVRGRLVQYRGEVQAMLGQSTEELRVRLASHLRKLRKRLLRDADDLQKRLAVYQAGAREGA

112

ERGLSAIRERLGPLVEQGRVRAATVGSLAGQPLQERAQAWGERLRARMEEMGSRTRDRLDEVKEQVAEVRAKLEEQAQQI

RLQAEAAQARLKSRFEPLAEDMQRQWAGQVEKVQAAEGTS AAPVPSDNH

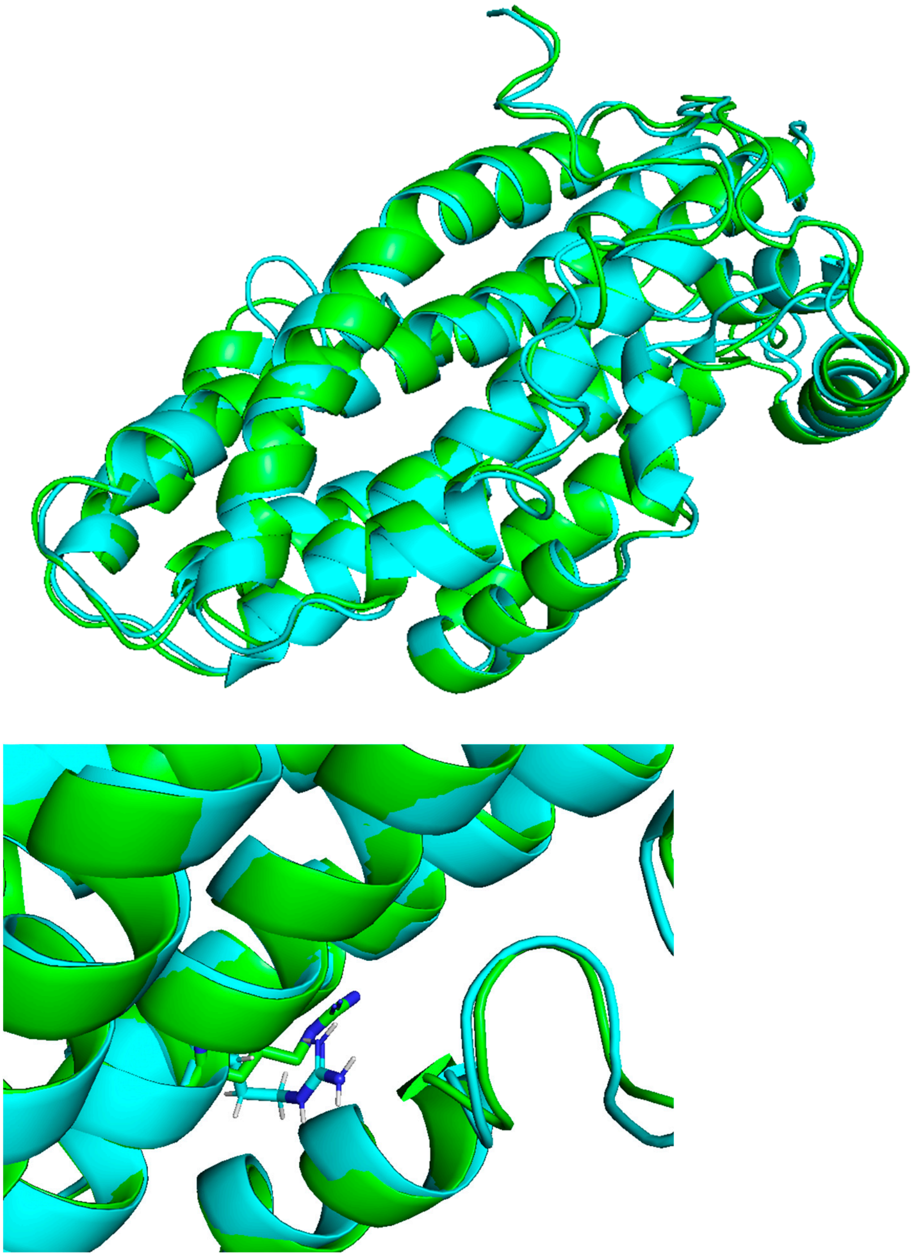

Fig.S7 The green and blue structures represent the ApoE4 models obtained using PyMOL and Swiss Model, respectively. To ensure a more accurate model, we used both software tools for comparison. Since the resulting structures were highly similar, the model with the best performance across various metrics was selected as the simulation target to ensure structural validity.

	33	34	35	36	37	38	39	40	41	42	43
(+)APOE4	0.1581	0.1536	0.1098	0.1212	0.1051	0.1285	0.1086	0.1346	0.1399	0.1148	0.1263
control group	0.1405	0.142	0.1013	0.1138	0.0916	0.1183	0.0985	0.1252	0.1299	0.1016	0.1121
(+)APOE3	0.1529	0.1321	0.1019	0.1149	0.0973	0.1067	0.1014	0.1284	0.1277	0.1062	0.1127

	47	48	49	50	51	52	53	54
(+)APOE4	0.223	0.131	0.1058	0.1253	0.1419	0.1378	0.1267	0.1262
control group	0.2118	0.1168	0.0928	0.1173	0.1286	0.1395	0.1131	0.1161
(+)APOE3	0.2096	0.1175	0.0929	0.1148	0.1252	0.1423	0.1142	0.1129

	61	62	63	64	65	66	67	68	69
(+)APOE4	0.1254	0.1486	0.1104	0.129	0.1352	0.1195	0.095	0.1296	0.0927
control group	0.1151	0.1377	0.0937	0.1152	0.1252	0.1116	0.0794	0.1179	0.0843
(+)APOE3	0.117	0.1431	0.096	0.1125	0.1267	0.119	0.0775	0.1164	0.0846

	79	80	81	82	83	84	85
(+)APOE4	0.1342	0.1117	0.1146	0.1367	0.1709	0.1671	0.1785
control group	0.1135	0.0916	0.0904	0.108	0.1436	0.1337	0.1522
(+)APOE3	0.1248	0.0964	0.0982	0.1241	0.1549	0.1396	0.1699

	210	211	212	213	214	215	216	217	218	219	220
(+)APOE4	0.2051	0.1511	0.1282	0.102	0.1428	0.1239	0.1465	0.1349	0.1412	0.1304	0.1094
control group	0.1937	0.141	0.1128	0.0916	0.1323	0.1027	0.0961	0.1207	0.1272	0.1116	0.0983
(+)APOE3	0.1993	0.1377	0.1054	0.0931	0.1335	0.1021	0.1006	0.1218	0.1303	0.1178	0.1051

	225	226	227	228	229	230
(+)APOE4	0.134	0.112	0.1126	0.1669	0.151	0.1314
control group	0.1188	0.0979	0.0973	0.1549	0.1388	0.1163
(+)APOE3	0.1237	0.1018	0.1033	0.1586	0.1394	0.1244

	240	241	242	243	244	245	246	247	248
(+)APOE4	0.0751	0.1088	0.1212	0.1239	0.0792	0.1832	0.1664	0.0845	0.1201
control group	0.0684	0.0895	0.1238	0.1133	0.0749	0.1367	0.1562	0.0757	0.1011
(+)APOE3	0.0729	0.1099	0.1394	0.1234	0.0909	0.152	0.1671	0.0888	0.1178

Fig.S8: where Box1 to Box7 denote regions with significant RMSF fluctuations under (+) APOE4 conditions, which correspond to the approximate residue ranges 33-43, 47-54, 61-69, 79-85, respectively, 210-220, 225-230, and 240-248, respectively.

In response to this question we consulted the information to answer in detail, due to the content is too much, we explain the reasons in this paper in the supplementary material, the content is as follows.

1. High-Quality Structural Data:

PDB ID: 1N7S represents a high-resolution SNARE complex structure, meaning it provides very precise atomic-level information. High-resolution structures often reveal better details of molecular interactions and spatial arrangements, helping to gain a deeper understanding of the function and mechanism of the SNARE complex.

2. Integrity and Representativeness of the Structure:

The 1N7S structure is a complete SNARE complex, including all three key components—VAMP2, SNAP25, and SYNTAXIN1. This makes it a comprehensive and representative structure, offering insights into how these three proteins work together to form a functional complex for neurotransmitter release. Other SNARE structures may only include partial complexes or localized regions, lacking a holistic view of its function.

3. Experimental Validation:

PDB ID: 1N7S was determined through X-ray crystallography and is supported by experimental data. This method provides high-confidence data, ideal for understanding how molecules function within biological systems. As such, this structure is widely recognized in experimental studies, ensuring its reliability.

4. Extensive Literature Support and Application:

The 1N7S structure has been cited in numerous studies, particularly in the investigation of how the SNARE complex regulates neurotransmitter release. This makes it the preferred structure for researchers in this field, with extensive literature supporting its use in various experimental designs.

In summary, PDB ID: 1N7S is chosen because it provides a high-resolution, comprehensive, and experimentally validated model of the SNARE complex, which is crucial for understanding its role in neurotransmitter release and its involvement in the pathophysiology of Alzheimer's disease and other neurodegenerative disorders.

Before performing molecular docking, we carried out the following steps to ensure the structural integrity of the APOE4 protein. These steps help remove any structural inconsistencies that could affect the docking results, ensuring that the protein structure used in the simulations is reasonable and stable. The specific steps are as follows:

1. Eliminating Alternative Conformations

Open SPDBV and load the APOE4 protein PDB file. Check the 299 amino acid residues of APOE4 for alternative conformations (if present). In SPDBV, select the most suitable conformation (either by minimizing energy or selecting the most likely conformation). Delete any unnecessary alternative conformations and retain the chosen one.

2. Adjusting Terminal Residues

In SPDBV, inspect the N-terminal and C-terminal residues of APOE4. Use the "Build Side Chain" module to adjust the conformations of the terminal residues, ensuring their proper geometry. Add appropriate hydrogen atoms at the terminal positions and ensure the side chains are properly oriented.

3. Correcting Bond Orders

Open the APOE4 PDB file in SPDBV and check the bond orders. Correct any errors, including improper double or single bond assignments. If any chemical bonds are missing or structures are incomplete, manually correct these issues and optimize them

using SPDBV's functions.

4. Energy Minimization

In SPDBV, select the "Minimize" function to perform energy minimization. Set appropriate parameters (such as maximum iteration number and maximum energy change) and initiate the minimization process. After minimization, verify the structure to ensure the overall stability and reasonable geometry of the protein.

5. Final Verification

Ensure that the APOE4 protein does not exhibit significant geometric issues, such as overlapping atoms or abnormal bond angles. Save the final version of the PDB file, naming it according to your preference; in this case, it is named "APOE4_docking.pdb."

Summary:

These steps were performed to ensure the structural integrity of the target protein, APOE4, and to provide a suitable starting structure for subsequent molecular docking analysis. By following these procedures, we were able to improve the accuracy and reliability of the molecular docking results. (The processing steps may vary depending on the specific amino acids of each protein.)

RMSD summary statistic	Mean	Standard Deviation	Median	Min	Max
(+)APOE3	0.2355713	0.0181599	0.2333762	0.1841002	0.3130167
(+)APOE4	0.2596659	0.0189927	0.2581518	0.2038882	0.3422541
